# Chemical Modification of Influenza CD8+ T-Cell Epitopes Enhances Their Immunogenicity Regardless of Immunodominance

**DOI:** 10.1371/journal.pone.0156462

**Published:** 2016-06-22

**Authors:** Sietske K. Rosendahl Huber, Jolien J. Luimstra, Josine van Beek, Rieuwert Hoppes, Ronald H. J. Jacobi, Marion Hendriks, Kim Kapteijn, Casper Ouwerkerk, Boris Rodenko, Huib Ovaa, Jørgen de Jonge

**Affiliations:** 1 Centre for Infectious Disease Control (Cib), National Institute for Public Health and the Environment, Bilthoven, the Netherlands; 2 Division of Cell Biology, Netherlands Cancer Institute, Amsterdam, the Netherlands; 3 Institute for Chemical Immunology (ICI), Utrecht, the Netherlands; University of Alabama at Birmingham, UNITED STATES

## Abstract

T cells are essential players in the defense against infection. By targeting the MHC class I antigen-presenting pathway with peptide-based vaccines, antigen-specific T cells can be induced. However, low immunogenicity of peptides poses a challenge. Here, we set out to increase immunogenicity of influenza-specific CD8^+^ T cell epitopes. By substituting amino acids in wild type sequences with non-proteogenic amino acids, affinity for MHC can be increased, which may ultimately enhance cytotoxic CD8^+^ T cell responses. Since preventive vaccines against viruses should induce a broad immune response, we used this method to optimize influenza-specific epitopes of varying dominance. For this purpose, HLA-A*0201 epitopes GILGFVFTL, FMYSDFHFI and NMLSTVLGV were selected in order of decreasing MHC-affinity and dominance. For all epitopes, we designed chemically enhanced altered peptide ligands (CPLs) that exhibited greater binding affinity than their WT counterparts; even binding scores of the high affinity GILGFVFTL epitope could be improved. When HLA-A*0201 transgenic mice were vaccinated with selected CPLs, at least 2 out of 4 CPLs of each epitope showed an increase in IFN-γ responses of splenocytes. Moreover, modification of the low affinity epitope NMLSTVLGV led to an increase in the number of mice that responded. By optimizing three additional influenza epitopes specific for HLA-A*0301, we show that this strategy can be extended to other alleles. Thus, enhancing binding affinity of peptides provides a valuable tool to improve the immunogenicity and range of preventive T cell-targeted peptide vaccines.

## Introduction

For many infectious diseases, cellular responses are required for clearance of the pathogen from the host. One such disease that causes serious health threats worldwide is influenza [1]. Preventive influenza vaccines mainly confer protection via antibodies directed against the highly variable surface proteins hemagglutinin (HA) and neuraminidase (NA). Influenza virus can escape previously induced immunity due to mutations in antigenic sites, so-called antigenic drifts. Consequently, protection is subtype or strain-specific and regular vaccine updates are required. In addition, current vaccines do not provide protection against newly emerging influenza subtypes, which has led to pandemics four times in the last century and most recently in 2009 [2, 3]. Cellular responses are often directed towards more conserved parts of the virus and may therefore provide cross-protection; however, eliciting these responses by vaccination remains a challenge [4, 5]. Vaccination with peptides that target antigen-specific T cells is one of the approaches that could induce these cross-protective cellular responses [6].

In general, peptide vaccines may aid in treating or preventing various types of diseases [7]. Kenter et al. reported a therapeutic cancer vaccine based on long overlapping peptides that induced robust T cell responses leading to clinical effectiveness [8]. Over the past years, preclinical research and two phase I clinical trials were reported, in which preventive influenza vaccines containing a set of long overlapping peptides capable of inducing T cell responses were described [9–11]. Whether or not a peptide is capable of inducing such responses is dependent on characteristics such as length of the peptide and adjuvation. The latter is required, since peptides alone are often weak immunogens [12]. We recently described a method to increase immunogenicity of peptides in the context of therapeutic anti-tumor vaccination, by substitution with amino acids that are not naturally incorporated into proteins, so-called non-proteogenic amino acids [13]. By expanding the natural protein code, we aimed to generate peptides that increase peptide-MHC binding more than achieved by using substitution with proteogenic amino acids. The resulting chemically enhanced peptide ligands (CPLs) had increased binding affinities compared to the wild type peptides, which in turn led to enhanced T cell responses. Here, we used this approach to modify peptides encoding highly conserved influenza-specific class I epitopes of varying dominance in the immune response to influenza infection. This approach could ultimately be used for a preventive influenza vaccine.

Individuals with preexisting cytotoxic influenza-specific T cells were shown to have an immunological advantage upon encounter with influenza virus due to cross-reactivity of these T cells [14–16]. The presence of cross-reactive cytotoxic T cells has even been shown to limit disease [17]. Several preventive short (9–10 aa) peptide vaccination concepts, focusing on highly conserved CD8^+^ T cell-specific influenza peptides, have been described [18–21]. Immunogenicity of these peptide vaccines was enhanced by methods such as incorporation of peptides into virosomes or liposomes and ligation of the peptides to a lipid tail. These methods proved promising in mouse experiments. However, these approaches were aimed at increasing immunogenicity by adding adjuvants or by using different modes of delivery, but none increased intrinsic immunogenicity of the peptides.

Immunogenicity of a peptide is defined by three interacting partners: peptide, MHC and TCR [22]. Class I peptides are generated during degradation of a protein by the proteasome, followed by loading of the peptides on MHC I molecules [23]. Each MHC allele has a different peptide-binding groove with specific binding pockets in which amino acid side chains of a peptide’s anchor residues can protrude [24, 25]. Amino acid positions of peptides are referred to as P_1_-P_C_, P_1_ being the N-terminal and P_C_ the C-terminal residue. By altering the anchor residues, which are usually found towards the C- and N-termini of the peptide, the number and/or quality of interactions between the peptide and MHC molecule can be altered, thereby increasing peptide affinity [26, 27]. This will result in prolonged presentation of peptides on the cell surface, which may lead to enhanced T cell immunogenicity [28, 29]. Modification of the central amino acids of the peptide, on the other hand, frequently results in abrogated T cell reactivity, since this part of the epitope is directly recognized by the TCR [30–32].

In this study, we focused on improving the binding affinity of short (9–10 aa) highly conserved influenza-specific epitopes in order to enhance their immunogenicity. We selected three highly conserved influenza epitopes specific for HLA-A*0201, the most abundant HLA allele in the Caucasian population, based on their varying binding affinities and dominance in influenza A virus infection: the highly dominant GILGFVFTL (M1_58-66_), the less dominant FMYSDFHFI (PA_46-54_) and the low affinity subdominant NMLSTVLGV (PB1_413-421_) epitopes[33]. We show that substitution with non-proteogenic amino acids can lead to improved HLA binding and T cell responses as measured by IFN-γ production in both in vitro and in vivo models. Moreover, we show that this strategy can be applied to epitopes specific for other alleles by improving binding of influenza epitopes ILRGSVAHK (NP_265-273_), SFSFGGFTK (PB2_322-330_) and RMVLSAFDER (NP_67-76_) (in order of decreasing dominance) to HLA-A*0301, another frequently occurring allele in the Caucasian population [34]. Thus, by enhancing binding affinity, responses to dominant and more importantly to otherwise subdominant epitopes can be improved.

## Materials and Methods

### Ethics Statement

This study was approved by the Committee on Animal Experimentation of the Netherlands Vaccine Institute (Bilthoven, the Netherlands) (permit numbers PO201200042, PO201200222) and the Committee on Animal Experimentation of the Antonie van Leeuwenhoek terrain (DEC-ALt) (permit numbers PO201300122, PO201400121, PO201400177 and PO201400188) (Bilthoven, the Netherlands). Animal handling was carried out in accordance with relevant Dutch national legislation, including the 1997 Dutch Act on Animal Experimentation. Mice were housed in filtertop macrolon III cages provided with cage enrichment (Igloo’s and nestlets). Mice were provided with SRM-A food (γ-irradiated, Arie Blok BV, the Netherlands) and tap water ad libitum and checked twice daily for their health condition. When possible, mice were anesthetized during handling by isoflorane in O2 to minimize suffering. The humane end point was defined as ruffled fur, inactive, cold and more than 20% of body weight loss. None of the animals reached the humane end point during any of the studies. When the experimental end point was reached, mice were anesthetized (isoflurane/O_2_), bled by orbital puncture and terminated by cervical dislocation.

### Peptide Design and Synthesis

Peptides were designed as described before and synthesized at the Netherlands Cancer Institute Peptide Facility by standard solid-phase peptide synthesis using Syro I and Syro II synthesizers [13]. The non-proteogenic amino acids were at first selected for their availability. Based on the results of the initial binding assays the set was narrowed down to those that increased binding. Using that knowledge the set was expanded with amino acids with similar side chain properties. Amino acids were purchased from Chiralix, NovaBiochem, Chem-Impex or Creo Salus. Resins were purchased pre-loaded with proteogenic amino acids (Nova Biochem) or loaded with non-proteogenic amino acids. Typically, 2-chlorotrityl chloride resin corresponding to a loading of 0.3 mmol (Nova Biochem) was swollen in dichloromethane (DCM, Biosolve); 0.15 mmol of amino acid and 0.51 mmol di-isopropylethylamine (DIPEA, Sigma-Aldrich) were added and the mixture was shaken for 10 minutes. Another 0.99 mmol DIPEA in DCM was added and the mixture was shaken for one hour. The reaction was quenched by addition of methanol. For large scale testing of binding affinity, peptides were synthesized on a small scale (2 μmol). Peptides selected for the in vitro functional assays were synthesized on a large scale (25–50 μmol) and purified by reversed-phase HPLC (Waters). Masses of all peptides were analyzed by LCMS (Waters) to confirm correct synthesis.

### Fluorescence Polarization-Based Peptide Binding Assay

Peptide-MHC affinity was measured using a fluorescence polarization (FP) assay based on UV-mediated ligand exchange [35–39]. Since the fluorescence emission of MHC-bound tracer peptide is polarized to a greater extent than that of non-bound tracer, the total FP is a measure for the ratio of bound versus unbound tracer peptide. MHCs were refolded with conditional ligand KILGFVFJV for HLA-A*0201 and RIYRJGATR for HLA-A*0301, in which J is the photocleavable 3-amino-(2-nitrophenyl)propionic acid. Soluble MHC was dissolved in PBS containing 0.5 mg/ml bovine γ-globulin (BGG, Sigma-Aldrich) to a final concentration of 0.75 μM. The HLA-A*0201 tracer peptide FLPSDCFPSV and the HLA-A*0301 tracer peptide KVPCALINK were fluorescently labeled at the cysteine residues with 5-N-maleimide tetramethylrhodamine. Tracer peptides were diluted to a concentration of 6 nM in 1×BGG/PBS. Peptides of choice were dissolved at 125 μM in DMSO. Using a Hamilton MicroLab Liquid Handling Workstation the components were automatically transferred in triplicate into a 384-well microplate (black polystyrene, Corning). MHC, tracer and peptide were combined to reach final concentrations of 0.5 μM, 1 nM and 4.2 μM, respectively. The plate was exposed to UV light (365 nm) for 30 minutes at 4°C to exchange the UV-sensitive peptide for the desired peptides. FP values were measured using a BMG PHERAstar plate reader. To generate IC_50_ curves the FP-based peptide binding assay was performed using serial peptide dilutions ranging from 224 nM to 4 μM. Data were analyzed using GraphPad Prism 5 software.

### IFN-γ Induction in a GILGFVFTL Specific T Cell Clone

TAP-deficient T2 cells, which are incapable of transporting peptides from the cytosol into the ER and thus only present exogenously loaded peptides, were cultured in RPMI 1640 medium (Invitrogen) supplemented with 10% FCS. The GILGFVFTL-specific T cell clone was cultured in RPMI 1640 medium containing 10% FCS supplemented with 3 U/ml IL-2. Per well of a 96-well plate, 50,000 T2 cells were pulsed with 10 pM of the desired peptides at 37°C for 1 hour. After washing away any unbound peptides, T2 cells were cultured in RPMI 1640 medium containing 10% FCS with 50,000 specific T cells for 24 hours in presence of 1 μl/ml Golgiplug (BD Biosciences). As positive control, T cells were stimulated with 0.05 μg/ml PMA (Sigma-Aldrich) and 1 μg/ml ionomycin (Sigma-Aldrich). Unstimulated cells were included as negative control. After incubation, the plate was centrifuged at 700 g for 2 minutes. The medium was discarded and cells were resuspended and stained with 20 μl/ml CD8-FITC antibody (BD Biosciences) in PBS with 0.5% BSA and 0.02% sodium azide). Cells were fixed and permeabilized using a Cytofix/CytoPerm kit (BD Biosciences) according to manufacturer’s recommendations. Then, cells were stained for intracellular IFN-γ using 20 μl/ml anti-IFN-γ-APC (BD Biosciences) and analyzed using a Beckman Coulter CyAn ADP flow cytometer. The percentage of IFN-γ^+^ cells was determined from the CD8^+^ gate. Data were analyzed using FlowJo version 7.6.1. software (Tree Star Inc).

### Isolation and Culture of Human DCs

PBMCs of HLA-A2-typed healthy human donors were isolated from fresh blood by gradient centrifugation using Lymphoprep (Nycomed). Next, monocytes, CD8^+^ T cells and then CD4^+^ T cells were magnetically purified using CD14, CD8 or CD4 antibody-labeled magnetic beads, respectively, using LS columns according to manufacturer’s recommendations (Miltenyi Biotec). Following elution from the columns, CD8^+^ T cells and CD4^+^ T cells were frozen in FCS (Hyclone) with 10% DMSO and stored at -80°C until further processing. CD14^+^ cells were plated in a concentration of 0.4*10^6^ cells/ml in DC culture medium (IMDM (GIBCO, Invitrogen) containing 1% FCS, 100 U/ml penicillin, 100 μg/ml streptomycin, 292 μg/ml glutamine (all Sigma), supplemented with 500 U/ml human GM-CSF (PeproTech) and 800 U/ml human IL-4 (Active Bioscience) and incubated for six days at 37°C.

### Maturation and Co-Culture of DCs

After six days of culture, half of the DC culture medium was replaced with DC culture medium containing GM-CSF only, and 1 nmol peptide per well was added. After an incubation period of one hour, 10 ng/ml E. coli LPS (Invivogen) was added to mature the DCs. After 48 hours, DCs were harvested and plated in a U-bottom 96-well plate in a concentration of 5*10^3^ cells/well in co-culture medium (AIM-V (GIBCO) containing 2% human AB serum (Sigma)). Samples of the DCs were collected for analysis of maturation markers by flow cytometry. Next, autologous CD8^+^ and CD4^+^ T cells were added to the DCs in a both in a10:1 ratio. After seven days of co-culture, cells were collected for analysis by flow cytometry.

### Flow Cytometry

To determine maturation status, DCs were harvested two days after addition of peptides and maturation factor LPS. Cells were stained in FACS buffer (PBS (GIBCO) containing 0.5% BSA (Sigma) and 0.5 mM EDTA (ICN Biomedicals)) for 30 minutes at 4°C with either one of two panels that contained the following maturation markers: anti-CD80-FITC, anti-CD14-PE, anti-DC-SIGN-APC, anti-HLA-DR-Pacific Blue and Live/dead-AmCyan (Invitrogen) (panel 1) or anti-CD83-FITC, anti-CD40-PE, (BD Biosciences), anti-PD-L1-APC (eBioscience), anti-CD86-Pacific Blue (BioLegend) (panel 2). Live/dead-AmCyan (Invitrogen) was included in both panels. For analysis of the co-culture, the following markers were used: anti-CD8-FITC (Sanquin), anti-CD3-PerCP, anti-TNFα-PE-Cy7, anti-IFN-γ-APC (BD Biosciences), anti-CD4-Pacific Blue (eBioscience) and Live/dead-AmCyan (Invitrogen). Four hours prior to staining, Brefeldin A (BD Biosciences) was added to the culture; then cells were stained using the Cytofix/Cytoperm kit from BD Biosciences according to manufacturer’s recommendations. Cells were measured using a FACS Canto II (BD Biosciences) and results were analyzed using FlowJo version 9.7.5 software. First, lymphocytes were gated, followed by gating of live cells, then CD3^+^ cells and finally CD8^+^ or CD4^+^ cells were placed in a quadrant with TNF-α^+^ or IFN-γ^+^ cells.

### Immunization of Mice

HLA-A2 transgenic mice, B6.Cg-Tg (HLA-A/H2-D)2Enge/J (Jackson Laboratory, USA), maintained in house, or C57BL/6 mice (Charles River, Germany) were vaccinated with the indicated peptides at their respective doses in a volume of 100 μl. Peptides were adjuvanted with Incomplete Freund’s Adjuvant (IFA) (1/1 (V/V)) and CpG (50 μg/mouse) by vortexing the mixture for 30 minutes. In all experiments, mice were subcutaneously vaccinated at days 0 and 21 in alternating flanks. Two weeks after booster vaccination, mice were sacrificed, spleens were excised and spleen cells were restimulated for 16 hours with WT peptide or CPL. Specific IFN-γ responses were assessed using an ELISpot assay.

### ELISpot Assay

IFN-γ ELISpot assays were performed according to the manufacturer’s protocol (U-Cytech). Spleens were homogenized and passed through 70 μm filters (BD Biosciences), washed with RPMI 1640 containing 10% FCS, 100 U/ml penicillin, 100 μg/ml streptomycin and 292 μg/ml glutamine and counted using a Casy cell counter (Roche). Cells were plated in a concentration of 4*10^5^ cells/well in an IFN-γ antibody-coated PVDF membrane plate (Millipore MSIP) and stimulated with 0.1 nmol/well of either WT peptide or corresponding CPL. After 16 hours of incubation spots were visualized according to the manufacturer’s protocol (U-Cytech) and counted using an A.el.vis reader (Sanquin).

## Results

### Optimizing HLA-A*0201 Binding Affinity of Influenza Epitopes

**Fig 1:**
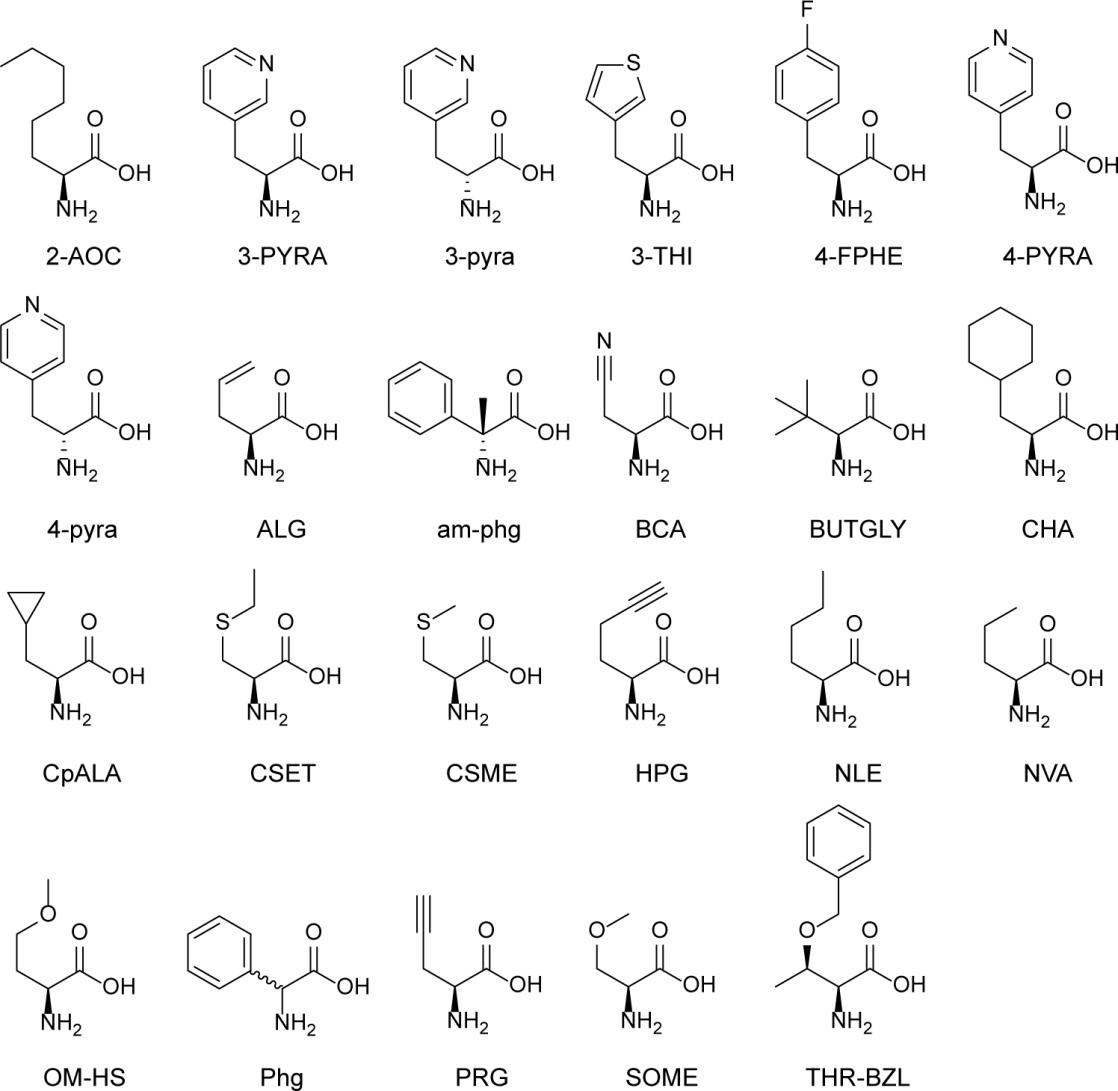
Structures of non-proteogenic amino acids found in the best CPLs. L-amino acids are denoted in uppercase characters; D-amino acids in lowercase characters. Incorporation of Phg results in a racemic mixture.

To enhance affinity for HLA-A*0201, amino acids of the WT peptides were substituted with non-proteogenic amino acids (Fig 1). Three influenza-specific epitopes were selected based on their varying binding affinity and dominance in the immune response. Per epitope, approximately 200 peptides were rationally designed based on available crystal structures and on side chain similarities. Binding affinity was determined by a fluorescence polarization (FP) assay (S1 Table), in which CPLs compete with fluorescent tracer peptide for HLA-A*0201 binding [35, 39]. From the difference in FP of MHC with tracer alone and in combination with CPL, the binding strength of the test peptide was scored as percentage inhibition of tracer peptide binding. This method allowed for high-throughput testing of multiple peptides. Per epitope, we selected 20 CPLs for their varying binding affinities ranging from the best binding CPLs to CPLs that bound approximately equally well as the WT peptide in order to study the correlation between binding scores and in vitro and in vivo responses (Table 1). After 4 hours, many of the peptides showed increased binding, but those peptides that still showed increased binding after 24 hours are likely to have a lower off-rate as a result of their higher affinity. As depicted in Table 1, the binding score of WT GILGFVFTL was 84% after 24 hours of incubation. Insertion of the non-proteogenic amino acid D-α-methyl-phenylglycine (am-phg) on P_1_, resulted in the most successful CPL with a binding score of 98% (G1; see Table 1). Other successful substitutions on P_1_ were mainly aromatic amino acids, such as DL-phenylglycine (Phg) (G7; see Table 1), or the L (represented in uppercase) and D (represented in lowercase) amino acids of 3'- and 4’-pyridyl-alanine (3- and 4-PYRA; 3- and 4-pyra;), which also resulted in increased binding scores (G8, G15, G4 and G10, Table 1).

**Table 1:** FP binding scores of selected HLA-A*0201 peptides.

A						B						C					
#	GILGFVFTL	4h	SD	24h	SD	#	FMYSDFHFI	4h	SD	24h	SD	#	NMLSTVLGV	4h	SD	24h	SD
G1	[am-phg]ILGFVFTL	97	4	98	4	F5	[4-FPHE]MYSDFHF[2-AOC]	95	2	94	2	N95	[4-FPHE][2-AOC]LSTVLGV	92	1	90	1
G7	[Phg]ILGFVFTL	96	0	94	2	F118	[CSET][2-AOC]YSDFHFI	95	4	93	3	N177	F[2-AOC]LSTVLGV	90	2	88	2
G8	[3-PYRA]ILGFVFTL	94	3	93	1	F141	[THR-BZL][2-AOC]YSDFHFI	94	2	93	2	N11	[am-phg]MLSTVLG[2-AOC]	90	2	88	2
G15	[4-PYRA]ILGFVFTL	93	3	93	3	F48	F[2-AOC]YSDFHF[CHA]	93	1	92	1	N91	[OM-HS][2-AOC]LSTVLGV	91	2	87	1
G4	[3-pyra]ILGFVFTL	92	6	92	6	F143	F[2-AOC]YSDFHFI	93	2	91	2	N169	[CSME][2-AOC]LSTVLGV	89	4	87	4
G12	GILGFV[4-FPHE]TL	92	3	92	5	F102	[3-THI][2-AOC]YSDFHFI	92	0	91	0	N172	[PHG][2-AOC]LSTVLGV	89	3	86	4
G27	[am-phg][CpALA]LGFVFTL	92	1	91	2	F112	[BCA][2-AOC]YSDFHFI	92	4	91	3	N8	[3-PYRA]MLSTVLG[2-AOC]	85	7	82	7
G10	[4-pyra]ILGFVFTL	90	2	91	4	F7	[am-phg]MYSDFHF[2-AOC]	91	4	90	4	N92	[SOME][2-AOC]LSTVLGV	84	3	81	3
G9	yILGFVFTL	94	2	90	2	F69	F[2-AOC]YSDFHF[NLE]	90	3	89	2	N98	[3-THI]MLSTVLG[2-AOC]	83	2	79	1
G16	G[NLE]LGFVFTL	91	3	90	5	F49	FMYSDFHF[CHA]	88	2	86	3	N46	[am-phg]MLSTVLGV	80	1	76	1
G3	GILGFVFT[CpALA]	90	5	90	5	F19	FMYSDFHF[2-AOC]	87	2	86	2	N41	NM[2-AOC]STVLGV	78	5	73	5
G25	[SOME]ILGFVFTL	89	12	89	13	F193	[am-phg][NVA]YSDFHFI	85	3	81	2	N176	[THR-BZL]MLSTVLGV	73	3	70	3
G13	GILGFVFT[ALG]	88	8	88	7	F54	FMYSDFHF[CSET]	82	4	80	4	N40	N[2-AOC]LSTVLGV	71	6	67	5
G17	GILGFVFT[PRG]	88	3	88	5	F95	[2-AOC]MYSDFHFI	82	3	80	3	N15	[NVA]MLSTVLG[2-AOC]	71	11	65	10
**G WT**	**GILGFVFTL**	85	0	84	2	F105	[4-FPHE]MYSDFHFI	86	1	78	1	N39	[2-AOC]MLSTVLGV	68	8	63	7
G24	fILGFVFTL	86	8	81	7	F52	FMYSDFHF[CpALA]	81	3	78	4	N53	N[NLE]LSTVLGV	66	3	61	3
G20	GILGFV[BUTGLY]TL	82	10	79	12	F63	FMYSDFHF[HPG]	81	7	78	5	N122	NMLSTVLG[CpALA]	65	2	60	1
G22	GILGFVFT[2-AOC]	79	14	77	15	F142	[THR-BZL]MYSDFHFI	80	2	77	3	N61	[SOME]MLSTVLGV	62	1	54	7
G11	[CSME]ILGFVFTL	72	28	70	32	**F WT**	**FMYSDFHFI**	78	3	75	2	N52	[NLE]MLSTVLGV	54	4	46	5
G26	G[2-AOC]LGFVFT[PRG]	60	4	54	2	F100	[3-PYRA]MYSDFHFI	79	7	73	5	**NWT**	**NMLSTVLGV**	53	5	46	4
G29	[3-PYRA]ILGFVFT[2-AOC]	44	7	38	5	F111	[am-phg]MYSDFHFI	75	9	72	9	N43	[3-PYRA]MLSTVLGV	47	19	41	20

HLA-A*0201 binding of CPLs of influenza epitopes; (A) dominant GILGFVFTL (M1_58-66_), (B) subdominant FMYSDFHFI (PA_46-54_) and (C) subdominant NMLSTVLGV (PB1_413-421_), was determined using an FP-based competition assay. Binding was scored as percentage inhibition of tracer peptide binding after 4h and 24h in three independent experiments. Maintained binding after 24 hours indicates a lower off-rate, presumably due to increased stability. This table shows binding scores for the WT epitopes (bold) and 20 CPLs that were selected for in vitro and ex vivo testing. Peptides in italic were used in vaccination experiments. SD: standard deviation. See S2 Table for a heat map representation.

Since the two less immunodominant influenza epitopes FMYSDFHFI and NMLSTVLGV naturally have lower affinities compared to GILGFVFTL, we expected an even larger improvement for CPLs derived from these peptides. Substitution with the aromatic 4-fluorophenylalanine (4-FPHE) in combination with a substitution with L-2-amino-octanoic acid (2-AOC) resulted in CPLs with the highest binding score for both FMYSDFHFI and NMLSTVLGV epitopes. The binding score for FMYSDFHFI was raised from 75% to 94% after substitution of P_1_ with 4-FPHE, in combination with 2-AOC on P_9_ (F5; Table 1). 4-FPHE on P_1_ in combination with 2-AOC on P_2_, increased the binding score of NMLSTVLGV from 55% to 92% (N95; Table 1). Apart from these peptides, 2-AOC alone led to increased binding when substituted at or near the anchor positions P_2_ and P_9_ for both FMYSDFHFI and NMLSTVLGV (F143, F19, F95, N39, N41, N40; see Table 1). Thus, using non-proteogenic amino acid substitutions, we were able to increase the binding of peptides such that they nearly inhibited 100% of the tracer peptide from binding, regardless of the affinity of the WT epitope.

### In Vitro and Ex Vivo T Cell Activation Screening Assays

Since modifications could change the T cell-exposed peptide side chains in such a way that they do not resemble those of the WT peptide anymore, we investigated whether CPLs were still capable of activating WT-specific T cells. To determine this for modifications of GILGFVFTL, antigen-presenting T2 cells were pulsed with CPLs and co-cultured with a GILGFVFTL-specific T cell clone. Subsequently, IFN-γ production was determined by flow cytometry after 24 hours of culture. Approximately half of the 16 tested CPLs showed higher IFN-γ responses compared to the WT epitope (S3 Table). After 24 hours, G1 and G7, the CPLs with the highest binding affinity induced high IFN-γ responses. In addition, G16 and G25 with moderately improved binding affinity also induced high IFN-γ responses; however, 4 out of 13 CPLs with similar or improved binding showed strongly reduced to no activation. Therefore, affinity is to a certain extent indicative for CD8^+^ T cell activation, but fails as a predictor in some cases. The latter may indicate that the T cell-exposed peptide structure is altered.

Since T cell clones for FMYSDFHFI and NMLSTVLGV were not available, other assays were developed to allow pre-selection for in vivo experiments. To be able to compare the predictive value of these assays with that of the T cell clone-based assay, we also performed these assays with GILGFVFTL CPLs. The first alternative strategy included testing responses following CPL stimulation in a human HLA-A2^+^ DC T cell co-culture model. For this purpose, HLA-A2^+^ donors were selected based on the presence of CD8 specific IFN-γ responses after stimulation with WT peptide. Monocytes from these donors were isolated, differentiated into immature DCs and subsequently pulsed with different CPLs. After pulsing, DCs were matured and co-cultured with autologous T cells for seven days. Then, IFN-γ production of CD8^+^ T cells was measured by flow cytometry. Several CPLs appeared to induce a higher response than their corresponding WT peptides (S3 Table); however, this assay had both a high assay variation and a high variation between donors.

To limit inter-individual variation, a third strategy was developed, in which CPLs were tested ex vivo on splenocytes of HLA-A2 tg mice vaccinated with either one of the three WT epitopes. Two weeks post booster vaccination, spleen cells were isolated and restimulated for 16 hours with selected CPLs and IFN-γ levels were measured by ELISpot. In this assay, only CPLs G13 and F100 induced similar responses compared to their corresponding WT peptide (S3 Table). In general, the positive results of the three assays correlate poorly, as shown in Table 2 for the upper three CPLs after ranking the results based on T cell activation for each assay. However, a correlation between the three assays was found for the lower ranked CPLs derived from GILGFVFTL and NMLSTVLGV, which allowed for negative selection. We therefore used both positive and negative results from all assays to include or exclude CPLs for further investigation.

**Table 2:** Summary of pre-selection experiments.

	GILGFVFTL			FMYSDFHFI		NMLSTVLGV	
	T cell clone	DC model	Mouse splenocytes	DC model	Mouse splenocytes	DC model	Mouse splenocytes
**Upper 3**	G1	G26	G13	F49	F100	N172	N92*
	G16	G7*	G3*	F5	F102*	N169	N40*
	G25	G15*	G22*	F54	F143*	N41	N172*
**Lower 3**	G4	G24	G24	F69	F49	N11	N15
	G9	G17	G9	F19	F5	N46	N11
	G24	G20	G4	F102	F7	N8	N8

* Lower response than WT peptide

CPLs were analyzed for their capacity to induce a response in WT-specific T cells. Therefore, three assays were developed in which IFN-γ production was used as a measure of response. The first was analysis of GILGFVFTL-CPLs on a WT-specific T cell clone (T cell clone). However, no T cell clone was available for the two other WT epitopes. Therefore, in the second assay, CPLs were loaded onto DCs of HLA-A2^+^ human donors and co-cultured for seven days with autologous CD8^+^ T cells (DC model). Due to high variation in the DC model, another assay was performed by 16 hours stimulation of splenocytes of WT-vaccinated HLA-A*0201 mice with CPLs (mouse splenocytes). This table shows the upper three and lower three CPLs after ranking the results based on T cell activation for each assay separately. * indicates when a CPL induced a response lower than that of the WT peptide control. A correlation between assays was found for the lower three CPLs derived from GILGFVFTL and NMLSTVLGV in all three assays. However, no correlation was found between assays for the upper CPLs.

### In Vivo Stimulation Using Modified Peptides

Vaccination of HLA-A2 tg mice with either of the three WT epitopes confirmed their dominance in the immune response as shown by the corresponding induction of IFN-γ as measured by ELISpot (S1A Fig). Since the HLA-A2 tg mice had a C57BL/6 background and co-expressed H2-Kb, a control experiment in C57BL/6 mice was performed. In these mice, no responses to the selected WT HLA-A*0201 epitopes were observed, which confirmed that responses in the HLA-A2 tg mice were HLA-A*0201-specific (S1A Fig). Subsequently, four CPLs per epitope were selected for in vivo testing. GILGFVFTL CPLs were selected based on binding scores and T cell clone data. To analyze a broad spectrum, CPLs with varying binding scores were selected (Table 1). Of these CPLs G1, G16 and G25 induced highest responses in the T cell clone, while G8 induced a response similar to that of the WT epitope (S3 Table).

**Fig 2:**
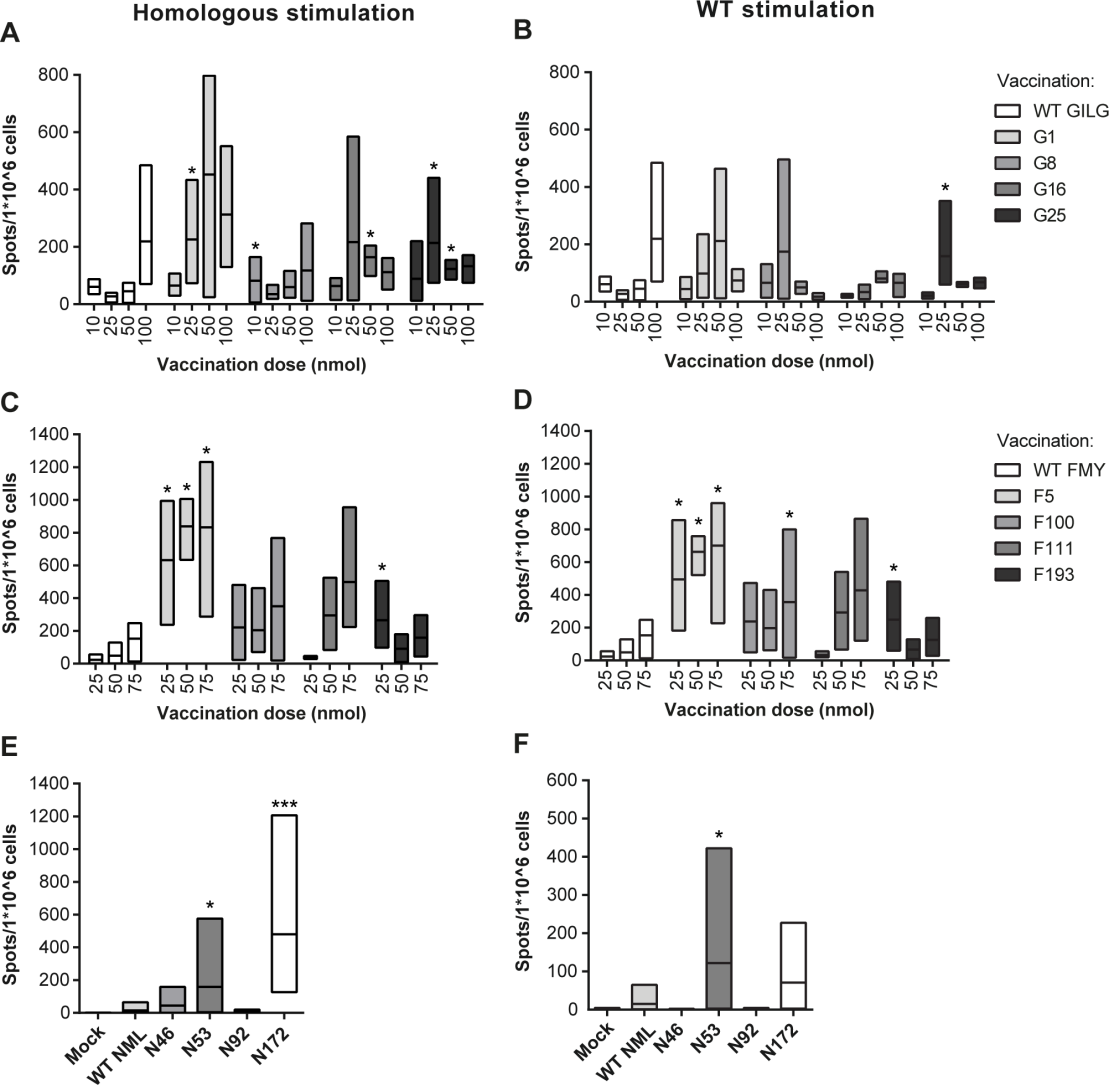
Vaccination with CPLs shows enhanced IFN-γ responses in vivo compared to vaccination with WT peptide. Mice were vaccinated with different doses of WT peptide or CPLs on day 0 and day 21 and two weeks later spleen cells were isolated and restimulated with homologous peptides or WT peptide. Responses were measured by IFN-γ ELISpot after 16 hours stimulation with 0.1 nmol peptide/well. Mice were vaccinated with mock (not shown), 10, 25, 50 or 100 nmol of WT GILGFVFTL or with the indicated CPLs. Spleen cells were restimulated with homologous **(A)** or WT **(B)** peptide. Overall, responses were highest after stimulation with CPL G1. For FMYSDFHFI mice were vaccinated with mock (not shown), 25, 50 or 75 nmol of WT peptide or the indicated CPLs. Cells were restimulated for 16 hours with homologous **(C)** or WT **(D)** peptide. Three out of four CPLs (F5, F100 and F111) induced higher responses compared to WT-peptide vaccination. For NMLSTVLGV mice were mock vaccinated or vaccinated with a dose of 75 nmol of WT peptide or respective CPLs. Spleen cells were restimulated with homologous **(E)** or WT **(F)** peptide. CPL N172 induced most T cells that responded to homologous stimulation, whereas N53 induced most T cells responding to WT peptide. Mock-vaccinated mice in experiments shown in Fig 2A-2D showed comparable responses to mock-vaccinated mice in Fig 2E-2F. Fig 2A-2D depict three mice per dose. Data in Fig 2E and 2F are derived from 7–8 mice per group, with the exception of the mock, for which three mice were included. Bars are min to max, with line at mean. Data were statistically analyzed using a Mann-Whitney test. * p<0.05; *** p<0.001 compared to the WT equivalent.

HLA-A2 tg mice were vaccinated with different doses of WT GILGFVFTL peptide or CPLs G1, G8, G16 or G25 on days 0 and 21. Two weeks post booster vaccination, spleen cells were isolated and stimulated for 16 hours with different peptides and analyzed using an IFN-γ ELISpot assay. First, the effect of enhanced binding affinity on a T cell response was investigated using homologous peptide as a stimulus (Fig 2A). Overall, responses of G1-vaccinated mice were highest and those of G8-vaccinated mice lowest. Responses of G16- and G25-vaccinated mice, on the other hand, were highest at a vaccination dose of 25 nmol peptide and did not increase at higher doses. However, these CPL-specific T cells might not recognize the WT epitopes. Restimulation of splenocytes of CPL-vaccinated mice with WT peptide mimics a natural situation in which CPL-induced T cells respond to infection with a virus containing the WT epitope. As shown in Fig 2B, responses of WT-vaccinated mice were low at peptide vaccination doses of 10, 25 and 50 nmol, but increasing the dose to 100 nmol resulted in higher T cell responses. G1- and G8-vaccinated mice, on the other hand, showed a higher response compared to WT vaccinated mice at lower doses (Fig 2B). At a dose of 100 nmol the difference between CPLs and WT-peptide vaccinated mice was reversed, which might be due to overstimulation by CPLs at these high doses. Overall, G1 and G8 were the most promising GILGFVFTL CPLs as they resulted in the largest increase in responses after restimulation with WT peptide.

Selection of CPLs for the other two epitopes was more challenging, since data obtained using the different pre-selection strategies did not correspond well (Table 2 and S3 Table). We therefore selected CPLs based on data from vaccination experiments with GILGFVFTL CPLs in addition to the results of the screening assays. The final selection for FMYSDFHFI comprised F5 based on the DC co-culture model, F100 because it performed well in WT-specific mouse splenocytes and F111 and F193 based on favorable substitutions observed in pilot experiments with GILGFVFTL CPLs in mice. Mice were vaccinated with these CPLs using three doses of peptide, since in the previous experiment we observed minimal responses at the lowest dose used (10 nmol). Homologous peptide restimulation showed that vaccination with all four CPLs dramatically increased T cell responses compared to WT peptide (Fig 2C). When cells were restimulated with WT peptide three out of four CPL-vaccinated mice (F5, F100, F111) clearly showed higher IFN-γ responses than WT peptide-vaccinated mice (Fig 2D). One CPL (F193) only showed higher responses than WT peptide at a vaccination dose of 25 nmol. Thus, modification greatly enhanced T cell responses for three out of four peptides, even at low vaccination doses.

For the epitope NMLSTVLGV, CPLs N46 and N53 were selected based on modifications that were successful in previous in vivo experiments with GILGFVFTL, N92 because it was one of the few peptides that induced a response similar to that of WT peptide in WT-specific mouse splenocytes and N172 based on the DC co-culture data. Since NMLSTVLGV is a very low affinity epitope, these CPLs had, as expected, the largest improvement in binding score (Table 1). Earlier experiments indicated that the WT peptide induced responses only in approximately one out of six mice; therefore we chose to focus on just one vaccination dose and to increase the number of mice to seven or eight per group to assure that at least 1–2 mice responded to WT peptide vaccination. Fig 2E and 2F show that vaccination with CPLs N46, N53 and N172 increased the responses compared to vaccination with WT peptide, whereas N92-vaccinated mice did not respond to restimulation (Fig 2E and 2F). All of the N172-vaccinated mice (n = 7), half of the N53-vaccinated mice (n = 4) and four of the N46-vaccinated mice responded to homologous peptide restimulation. When spleen cells of N172-vaccinated mice were restimulated with WT peptide, half of these mice (n = 3) responded. For CPL N53 the number of responders remained stable (n = 4), while there were no responders for CPL N46. By modifying NMLSTVLGV, responses could be induced in a larger proportion of mice compared to WT peptide and these responses were higher in all cases, which is a major enhancement for this very subdominant peptide. CPL N172 was among the top binders, further showing a correlation between binding affinity and T cell reactivity.

### Detailed Analysis of the Most Immunogenic CPLs

For each of the three epitopes the most immunogenic CPL was selected for a more detailed analysis. To this extent, G1 and F5 were selected, since these CPLs induced highest and most robust responses after homologous and WT-peptide restimulation. N53 and N172 were selected since both peptides induced higher responses in a larger number of mice than WT peptide. However, first an additional control experiment was performed in C57BL/6 mice to confirm that CPL responses were HLA-A2-specific. Unexpectedly, CPL F5 induced responses in these non-transgenic mice. From this, we can conclude that part of the extent of the responses of F5 in the HLA-A2 tg mice is due to presentation of the F5 peptide on H2-Kb. However, the response of HLA-A2 tg mice is still substantially higher; therefore the improvement observed for F5 is at least in part mediated by HLA-A*0201 (S1B Fig).

**Fig 3:**
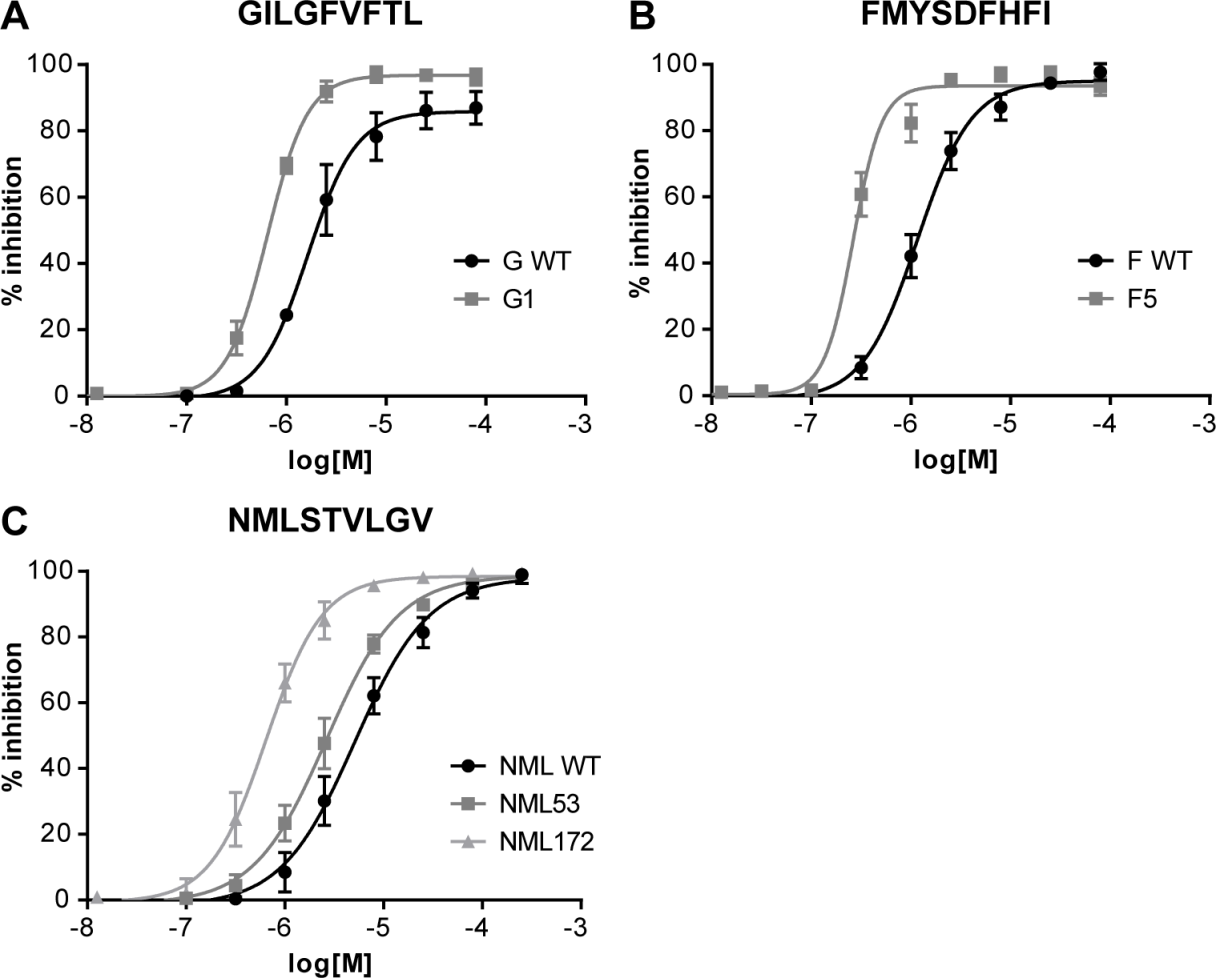
Binding affinity dose-response curves of CPLs and WT peptides. The IC_50_ curves of the selected CPLs show increased HLA binding affinity compared to IC_50_ curves of the corresponding WT-peptides. To generate IC_50_ curves the FP-based competition assay was performed using threefold peptide dilutions in the presence of a standard amount of tracer peptide. Shown are averages and their standard deviation of three independent experiments. Curves of CPLs are shifted to the left compared to WT peptides, indicating that a lower dose of CPLs is needed to inhibit tracer binding.

To provide more insight into binding affinity, serial peptide dilutions were used in the FP binding assay to determine the half-maximal inhibition of tracer binding concentration (IC_50_ values). Peptide binding scores as shown in Table 1 were determined at a single concentration. Analysis of the dose-response curves shows that in all cases the CPLs have a lower IC_50_ value than their WT counterparts (Fig 3) do. These results are in line with findings in vaccination experiments with mice, in which the GILGFVFTL- and FMYSDFHFI-derived CPLs induced an IFN-γ response at lower doses than the WT epitope (Fig 2). The increase in binding affinity probably not only results in an increased on-rate, but more importantly also a decrease in off-rate due to increased peptide-MHC (pMHC) stability [40]. This would cause a prolonged presentation to T cells and hence a higher IFN-γ response.

**Fig 4:**
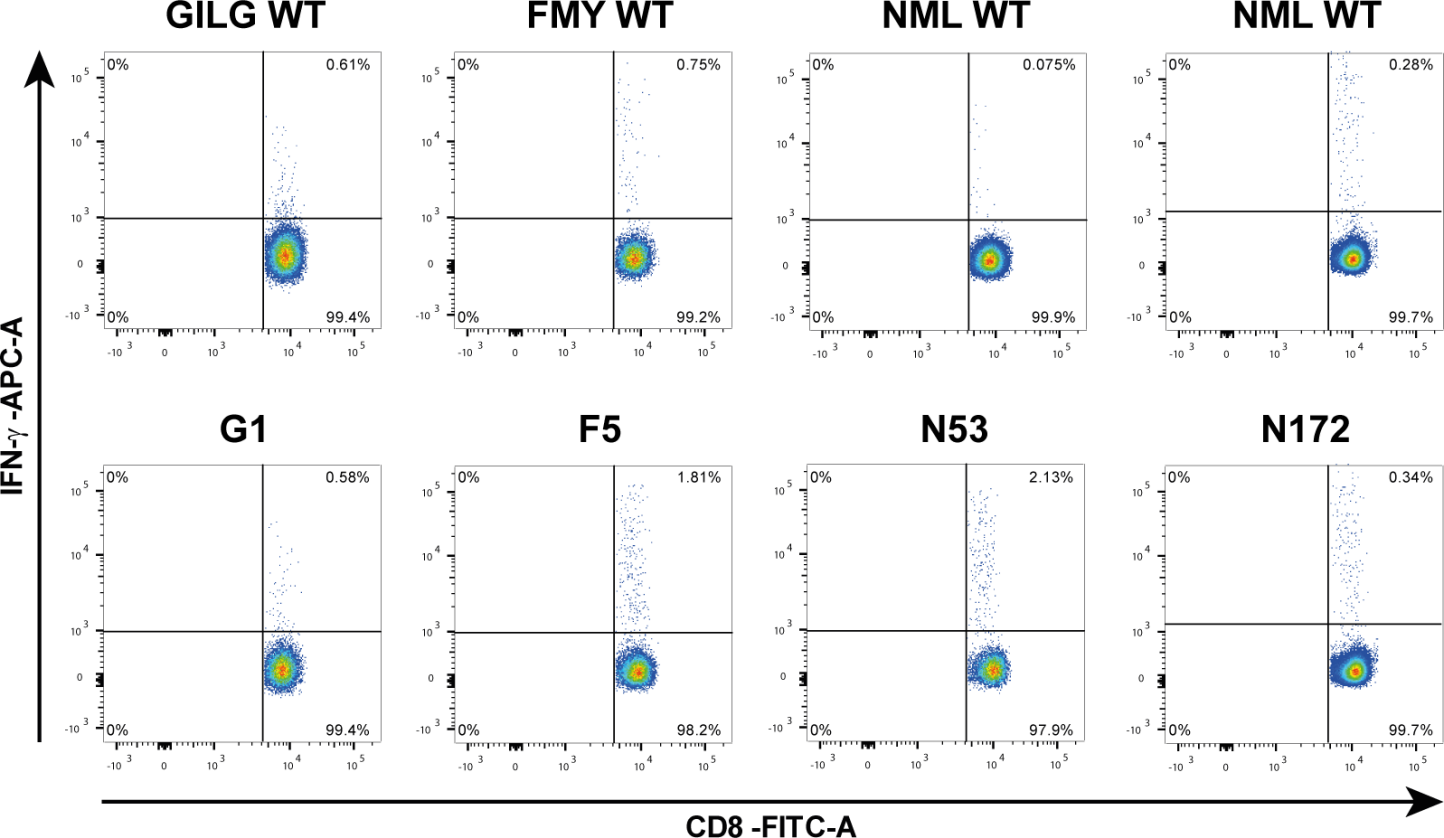
Flow cytometry analysis on CD8^+^ T cell responses of CPL- and WT-vaccinated mice. Dot plots showing IFN-γ production by CD8^+^ T cells of mice vaccinated with 75 nmol of either WT peptide or CPL (G1, F5, N53 and N172). In the upper panel, the respective WT-peptide control of that particular experiment is shown. In the lower panel, the CPL-induced IFN-γ responses are shown. Spleen cells (2*10^6^/well) were stimulated O/N with 1 nmol/well WT peptide. Highest responders of each group are shown. Vaccination with F5 and N53 induced the largest improvement in IFN-γ production compared to WT peptide-vaccinated mice. Negative control (mock stimulated splenocytes) had an average of 0.07% with a SD of 0.1%.

All responses obtained in the in vivo vaccination experiments were analyzed using an ELISpot assay with a complete pool of splenocytes. To prove that responses are indeed CD8^+^ T cell-specific, splenocytes were analyzed by flow cytometry. In Fig 4, flow cytometry dot plots show that the response towards CPL G1 was similar compared to WT peptide, which might be explained by the fact that a dose of 75 nmol was used. In the dose response experiments, a high dose of G1 appeared to result in suboptimal induction of IFN-γ production. Responses to CPLs F5 and N53, however, did show a major improvement as indicated by the increased production of IFN-γ by CD8^+^ T cells. CD4^+^ T cells did not produce IFN-γ in response to peptide restimulation, showing that the enhanced IFN-γ production measured in the ELISpot assay was produced by CD8^+^ T cells and not by CD4^+^ T cells (S2 Fig).

### Predictive Value of Modifications

**Fig 5:**
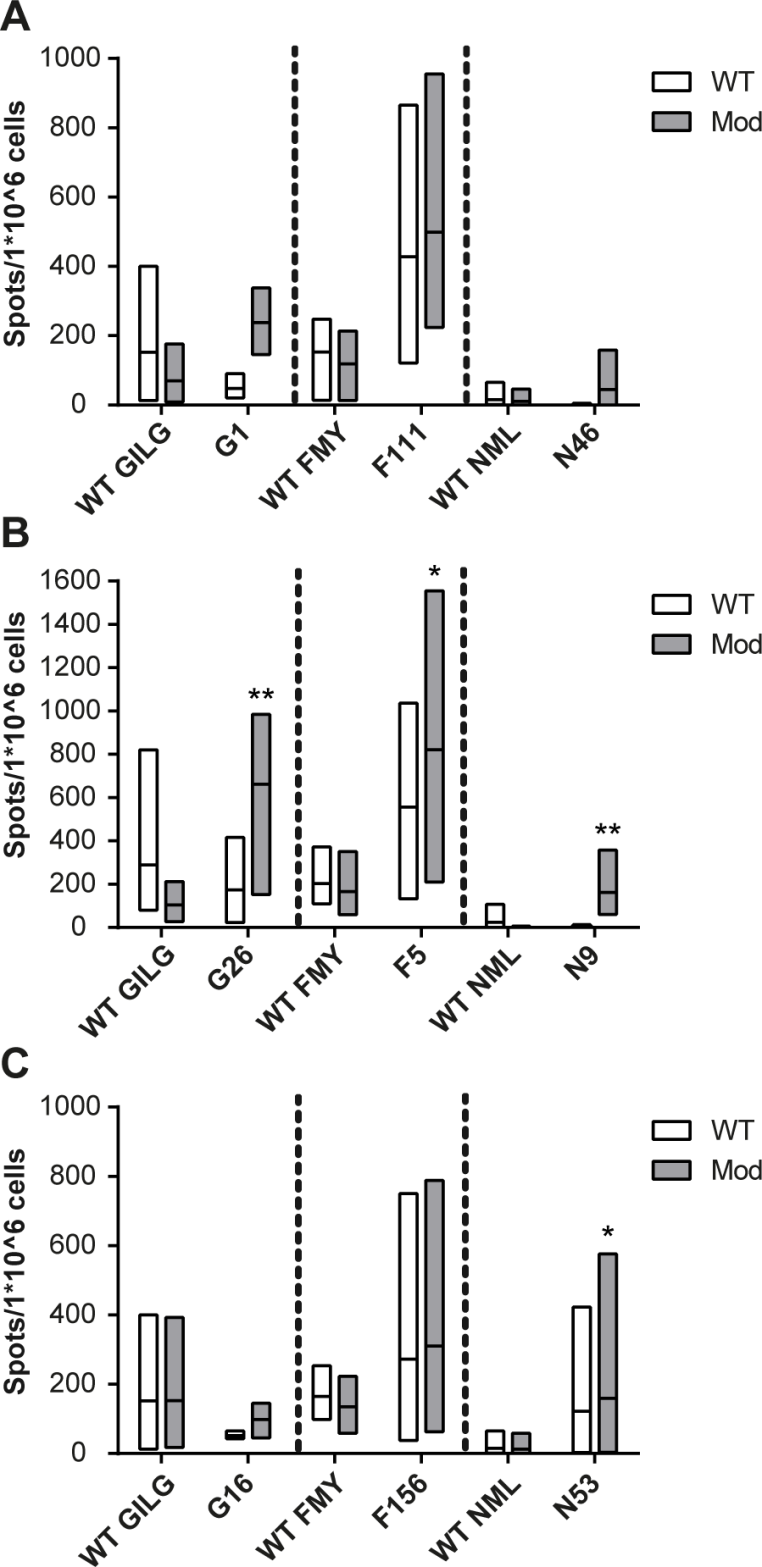
Predictive value of modifications. IFN-γ ELISpot on spleen cells of mice vaccinated with 75 nmol of either WT peptide or CPLs and stimulated for 16 hours with 0.1 nmol WT peptide or CPL per well. The three different modifications are based on final selected peptides for each epitope: **(A)** am-phg on P_1_ based on G1, **(B)** 4-FPHE on P_1_ and 2-AOC on P_9_ based on F5 and **(C)** NLE on P_2_ based on N53. X-axis depicts peptide used for vaccination. White boxes represents restimulation with WT peptide and grey boxes restimulation with CPL. Bars are min to max, with line at mean. Although it appears difficult to predict whether a modification will work in a certain epitope, an effective modification in one epitope is in some cases also effective in other epitopes. Bars represent a minimum of three mice (GILGFVFTL and FMYSDFHFI) and a maximum of eight (NMLSTVLGV). Data were statistically analyzed using a Mann-Whitney test. * p<0.05; ** p<0.01 compared to the WT equivalent.

Next, modifications of the CPLs described above were analyzed further to determine whether an effective substitution in one epitope is a prediction for the success of that particular substitution for other epitopes. For each epitope, CPLs were synthesized with modifications that are present in G1, F5 and N53, resulting in a set of three CPLs per type of modification. Fig 5A shows IFN-γ responses of mice vaccinated with either of the selected epitopes of which P_1_ was substituted for the residue am-phg, the modification that was most successful for GILGFVFTL (G1). Grey bars visualize that after stimulation with homologous peptides, enhanced responses were observed in all CPL-vaccinated mice. After WT stimulation, responses remained more or less similar, except for the response to N46, which was reduced to zero. Based on CPL F5 we introduced 4-FPHE on P_1_ and 2-AOC on P_9_ of GILGFVFTL and NMLSTVLGV. This combination of substitutions again led to a greatly enhanced response after restimulation with homologous peptide (Fig 5B). However, for both epitopes, responses after WT restimulation were lower in CPL-vaccinated mice compared to responses of WT-vaccinated mice. Perhaps by changing the amino acids the structure of these CPLs differed too much from the WT, such that specificity for the WT sequence was lost. Finally, we substituted P_2_ for norleucine (NLE) in GILGFVFTL and FMYSDFHFI based on CPL N53 (Fig 5C). This substitution showed a slightly enhanced response for CPL F156 compared to its WT counterpart, but led to decreased responses for CPL G16. Although it appears difficult to predict whether a modification will work in a given epitope, an effective modification in one epitope proves in some cases also effective in other epitopes.

### Optimizing HLA-A*0301 Binding Affinity of Influenza Epitopes

The response to vaccination can be broadened by selecting more HLA-A*0201 peptides, but even more so by targeting multiple alleles. We therefore set out to optimize three influenza epitopes specific for HLA-A*0301 as an example that incorporation of non-proteogenic amino acids is a strategy that can be extended to other alleles. The main difference between HLA-A*0201 and HLA-A*0301 is the preference of HLA-A*0301 for long positively charged residues on P_9_, demonstrated by the frequent occurrence of lysine and arginine on the C-terminal anchor position, whereas side chains of amino acids on P_2_ still dock into a hydrophobic pocket [41]. Using a HLA-A*0301-specific tracer peptide we performed the FP-based competition assay described in Materials and Methods for 96 peptides per epitope and measured binding after 4 and 24 hours [42]. Similar to HLA-A*0201, the selected epitopes vary in affinity, with ILRGSVAHK binding to the MHC with highest affinity and being the most dominant, and 10-mer RMVLSAFDER a low affinity epitope [33, 43, 44]. SFSFGGFTK is an intermediate HLA-A*0301 binder with unknown dominance. Substitution with non-proteogenic amino acids on or near anchor positions resulted in greatly enhanced binding, as shown in Table 3. Since HLA-A*0301 has a hydrophobic binding pocket at P_2_ just like HLA-A*0201, incorporation of norvaline (NVA) or 2-AOC on P_2_ resulted in increased binding scores. Substitutions on P_9_ did not enhance binding for any of the epitopes tested, probably because the lysine in the WT sequence forms strong ionic interactions that are hard to improve with the pool of amino acids tested. These data show that the technique of substituting amino acids by non-proteogenic amino acids to increase binding affinity can be applied to epitopes of other alleles, which is valuable for the development of broadly immunogenic vaccines.

**Table 3:** FP binding scores HLA-A*0301 peptides.

A						B						C					
#	ILRGSVAHK	4h	SD	24h	SD	#	SFSFGGFTK	4h	SD	24h	SD	#	RMVLSAFDER	4h	SD	24h	SD
I65	ILRGSV[2-AOC]HK	87	2	94	0	S70	S[NVA]SFGG[2-AOC]TK	90	0	96	0	R71	R[NVA]VLSAF[2-AOC]ER	71	2	82	2
I74	I[AHA]RGSV[NLE]HK	77	2	92	1	S84	SF[4-FPHE]FGGFTK	82	1	95	0	R67	R[AHA]VLSAF[2-AOC]ER	74	2	78	1
I53	[AHA][NLE]RGSVAHK	73	0	91	2	S50	S[NVA][2-AOC]FGGFTK	83	1	94	1	R81	RMVLSAF[AHA]ER	71	2	76	2
I87	I[AHA][4-FPHE]GSVAHK	73	2	91	0	S74	S[NVA]SFGG[CpALA]TK	78	1	94	1	R38	RMVLSAF[2-AOC]ER	69	3	75	2
I60	[2-AOC][NLE]RGSVAHK	72	4	91	1	S68	S[NVA]SFGG[NLE]TK	79	2	93	0	R70	R[NVA]VLSAF[SOME]ER	64	4	74	1
I89	I[AHA][CHA]GSVAHK	75	1	91	1	S95	S[NVA][4-FPHE]FGGFTK	80	2	93	0	R42	RMVLSAF[CpALA]ER	65	2	73	1
I59	I[AHA][2-AOC]GSVAHK	71	1	91	0	S94	S[NVA][NLE]FGGFTK	80	1	93	1	R36	RMVLSAF[NLE]ER	66	4	73	1
I73	I[AHA]RGSV[CSET]HK	74	3	90	1	S52	S[NVA][AHA]FGGFTK	80	1	93	1	R72	R[NVA]VLSAF[4-FPHE]ER	68	1	72	2
I61	[CSME][AHA]RGSVAHK	71	2	90	1	S72	S[NVA]SFGG[4-FPHE]TK	79	0	93	0	R83	RM[OrnN2]LSAFDER	71	3	72	2
I49	I[OrnN2]RGSVAHK	71	2	90	1	S93	S[NVA][CSME]FGGFTK	80	0	93	1	R4	RMVLSAFDEK	61	1	69	1
I79	I[AHA]RGSV[2-FUR]HK	77	3	90	1	S53	S[NVA]RFGGFTK	80	1	93	1	R59	R[NVA]VLSAFDKR	62	4	68	0
I86	I[AHA][CSME]GSVAHK	71	2	89	1	S54	S[AHA]RFGGFTK	80	1	92	1	R2	R[AHA]VLSAFDEK	58	1	67	1
I84	I[AHA][PRG]GSVAHK	71	2	89	0	S92	S[NVA]SFGGF[TOME]K	80	2	92	1	R50	RM[2-AOC]LSAFDER	70	3	66	1
I77	I[AHA]RGSV[CpALA]HK	71	2	89	1	S73	S[NVA]SFGG[BPG]TK	73	0	91	1	R55	RM[4-FPHE]LSAFDER	70	4	65	1
I63	ILRGSV[NLE]HK	74	2	89	1	S69	S[NVA]SFGG[NVA]TK	72	2	91	0	R68	R[AHA]VLSAF[4-FPHE]ER	67	3	64	0
I17	[2-AOC][AHA]RGSVAHK	61	5	89	2	S96	S[NVA][CpALA]FGGFTK	76	3	91	0	R66	R[AHA]VLSAF[SOME]ER	63	3	63	1
I58	I[AHA][NLE]GSVAHK	68	3	89	1	S56	S[NVA]SFGGFHK	78	2	91	1	R41	RMVLSAF[ORN]ER	65	3	63	0
I83	I[AHA][CpALA]GSVAHK	70	3	89	0	S76	S[NVA]SFGG[2-FUR]TK	75	1	91	0	R39	RMVLSAF[PRG]ER	62	4	62	1
I94	IL[4-FPHE]GSVAHK	70	4	89	1	S71	S[NVA]SFGG[PRG]TK	70	2	90	0	R37	RMVLSAF[CSET]ER	60	3	60	2
I85	I[AHA][SOME]GSVAHK	69	2	88	1	S90	S[NVA]SFGGF[AHA]K	78	0	90	1	R40	RMVLSAF[4-FPHE]ER	63	3	57	1
**I WT**	**ILRGSVAHK**	66	3	87	1	**S WT**	**SFSFGGFTK**	67	2	58	3	**R WT**	**RMVLSAFDER**	18	2	6	1

HLA-A*0301 binding data for three influenza epitopes: (A) ILRGSVAHK, (B) SFSFGGFTK* AND (C) RMVLSAFDER. Affinity was determined as in Table 1 after 4h and 24h in three independent experiments. This table shows percentage inhibition for the WT epitopes (bold) and 20 CPLs with highest binding scores. For all three epitopes binding scores could be greatly increased by substitution with non-proteogenic amino acids. See S4 Table for a heat map representation.

* SFSFGGFTK was incorrectly referred to in the immune epitope database; the epitope originally described as an HLA-A*0301 binder by Assarsson et al. has amino acid sequence: SFSFGGFTFK [43, 45].

## Discussion

Current vaccination strategies to prevent influenza infection are mainly aimed at antibody-mediated immune responses, yet cytotoxic responses have also been proven to contribute to protection against influenza infection [14, 16, 17, 46]. One of the approaches to induce these responses is by vaccination with peptides that encode T cell epitopes. However, immunogenicity of peptides is often inadequate; therefore, additional optimization is required. Here, we designed and synthesized CPLs with enhanced affinity for class I MHCs to improve, ultimately, T cell responses towards these peptides. Three highly conserved HLA-A*0201-specific influenza epitopes that have varying binding affinity and dominance in the immune response were selected: GILGFVFTL, a highly immunodominant epitope; FMYSDFHFI, a less dominant epitope, and NMLSTVLGV, which is a low affinity subdominant epitope. By studying available crystal structures and by replacing amino acids at or adjacent to the anchor positions with non-proteogenic amino acids, CPLs were designed with a theoretically increased number and quality of interactions with the MHC binding groove. Using non-proteogenic amino acids, modification was no longer limited to the repertoire of naturally occurring amino acids. With this approach, we succeeded to enhance binding affinity of all three epitopes and after in vitro evaluation, the most promising CPLs were tested in mice. We showed that CPLs G1, G8, F5, F100, F111 N53 and N172 were capable of inducing improved T cell responses in HLA-A2 tg mice, as measured by IFN-γ production in splenocytes. As expected, especially the response towards the more subdominant peptides was greatly improved.

The first objective was to improve binding affinity of the peptides to MHCs by introducing non-proteogenic amino acid substitutions. Earlier, we reported improved effectivity of a melanoma-specific peptide by substitution of am-phg on P_1_. This substitution led to additional interactions between the peptide and the MHC, thereby stabilizing the complex as shown in a crystal structure [13]. These findings may explain increased binding scores of CPLs G1 and N46, which contain the same substitution (Table 1). For FMYSDFHFI, introduction of am-phg on P_1_ retained the binding score at a similar level as WT peptide: (F111, Table 1). Surprisingly, G1 and F111, but not N46 showed improved immunogenicity in mice to the homologous and WT epitope (Fig 5).

Since the HLA-A*0201 allele prefers long hydrophobic residues on P_2_ and the C-terminus of a peptide, other stabilizing interactions were created by introducing hydrophobic residues into the peptide. 2-AOC, NLE and NVA are examples of amino acids with hydrophobic side chains that can protrude deeply into the hydrophobic binding pockets of HLA-A*0201 [47, 48]. CPLs of FMYSDFHFI and NMLSTVLGV with the largest increase in binding score indeed had a substitution of 2-AOC on P_2_ or P_9_, often in combination with other substitutions (Table 1). While introduction of 2-AOC did not enhance binding of GILGFVFTL-derived CPLs, introducing another hydrophobic residue, NLE, on P_2_ did enhance its binding score. In addition, this NLE substitution improved homologous immunogenicity of CPLs F156 and N53 and showed improved recognition of the WT epitope (Fig 5).

A point of interest is that from these binding results, it becomes clear that an amino acid preferred in one epitope is not necessarily preferred in another epitope, even when they are specific for the same HLA allele. Amino acid preferences are determined by the binding pockets in the binding groove of MHC and should therefore in theory be similar for every peptide specific for that allele. As discussed before, substitution of am-phg on P_1_ of the GILGFVFTL epitope resulted in the highest binding score (G1, 98% compared to 84% for the WT; Table 1) and a major improvement was seen for NMLSTVLGV after the same substitution on P_1_ (N46, 81% compared to 55% for the WT). The success of substitution on P_1_ is not surprising, since secondary anchor residues, which for HLA-A*0201 are found on P_1_, P_3_ and P_7_, were previously discovered to also have significant effect on binding [49, 50]. However, substitution of am-phg on P_1_ in the FMYSDFHFI epitope did not increase binding scores (F111, 72% compared to 75% for the WT, Table 1). Likewise, incorporating 3-PYRA on P_1_ was successful for the GILGFVFTL epitope (G8, 93%; Table 1), but did not enhance binding as much for NMLSTVLGV and FMYSDFHFI (both 73%; Table 1). This discrepancy could be due to conformational heterogeneity in the peptide backbones, since peptide binding strength is not only dependent on interactions of the side chains of anchor residues with the binding pockets, but also on those of the peptide backbone with the MHC binding groove [32, 51, 52]. The structure of the backbone is dependent on the size and fit of the amino acid side chains in the binding groove. Modifications may change the structure of the peptide backbone in one CPL in such a way that the interaction with the binding groove is weakened, while in another CPL there is no effect of the same substitution on this interaction. Alternatively, the change in structure of the backbone may affect the positioning of the anchor residue in such a way that it does not fit smoothly into the binding pocket.

Changes in the central region of the peptide may in turn affect recognition by the TCR [53, 54]. Thus, by introducing too many modifications in one peptide, T cell responses may be perturbed significantly and therefore we substituted a maximum of two amino acids. In addition, introduction of a single non-proteogenic amino acid in one peptide at a non T cell-exposed position might influence the structure of the backbone and thus the central region, while the same amino acid in another peptide might have little or no effect [55]. This could be the reason that some modifications always seem to lead to higher responses after restimulation with a CPL, likely due to the improvement of affinity, but that these CPL-induced T cells do not always react to restimulation with WT peptide (N46, G26, N9; Fig 5). These CPLs may induce a different subset of T cells than the WT peptide, which is not necessarily problematic in a vaccination setting as long as the CPL-induced T cells still recognize the WT peptide [56].

For the selection of CPLs for in vivo experiments, we set out to exclude CPLs that were not capable of inducing a response in WT-specific T cells as we hypothesized that these CPLs would likely not induce the correct T cells to recognize the WT epitope. Therefore, we performed three different assays in which CPLs were presented to WT-specific T cells. For the GILGFVFTL epitope a T cell clone was available, which facilitated analysis of responses of the WT-specific T cells to the CPLs. Activation of these cells by CPLs indicated that WT-specific TCRs are still capable of recognizing the CPLs (S3 Table). For FMYSDFHFI and NMLSTVLGV, other methods needed to be developed and we therefore included a human DC-T cell co-culture method and analysis of WT-specific mouse splenocytes stimulated by CPLs (S3 Table). The former analysis was effective in showing differences between the CPLs; however, donor variation was too large to draw definite conclusions. Analysis in splenocytes of an inbred HLA-A2 tg mouse strain allowed for little donor variation, but none of the CPLs were shown to induce better responses than the WT peptide in this model, in contrast to the other two methods. It did reveal some CPLs that induced little or no responses in the WT-specific splenocytes, allowing for negative selection. However, in these assays we were only able to mimic a reversed setting, i.e. WT-specific T cells that recognize CPLs. Such reverse immunology does not exclude the possibility that CPLs may induce T cells that are still capable of recognizing WT peptide even though this is not true for the inverted argument. For this reason, reverse immunology appears to be a suboptimal predictor for vaccine development [57]. Therefore, CPLs still needed to be tested for their ability to induce T cells that recognize the WT peptide in a vaccination setting.

Thus, we evaluated whether increased binding affinity also led to enhanced T cell responses by vaccination of HLA-A2 tg mice with a selection of CPLs (Fig 2). Based on results from the assays described above, four CPLs per WT peptide were selected for further in vivo testing. As our data for the GILGFVFTL-derived CPLs indicate, CPLs can facilitate a dose reduction while similar responses to WT peptide are maintained. At lower doses, vaccination with CPLs G1, G8 and G25 induced higher T cell responses after restimulation with WT peptide, compared to WT-vaccinated mice. The diminished responses at higher doses could be explained by overstimulation, as described for density of pMHC interactions on an APC [58]. In addition, modification of the FMYSDFHFI peptide led to the induction of higher T cell responses compared to the WT peptide in almost all doses tested. Surprisingly, F193 induced lowest homologous and heterologous responses, even though it did show improved binding affinity. In contrast, binding affinity of F100 and F111 were similar to that of the WT epitope, while these CPLs induced higher homologous and heterologous responses in mice. The effect of increasing binding affinity by introducing non-proteogenic amino acids on T cell responses was most remarkably shown by CPLs N53 and N172. These CPLs increased the number of responders to this subdominant epitope from approximately 1/6 to half of the mice and induced higher responses than the WT peptide, after WT restimulation. Hence, while increased binding may result in higher responses in mice, this appears not a general rule, which has some implications for vaccine development. Namely, the process to find modifications that lead to improved responses is not only affinity based, but also includes a trial and error factor. This may lengthen the development time of a peptide-based vaccine; however, with respect to the complete development process the impact is estimated to be minor.

Preventive vaccines should, most of all, induce a broad immune response, in contrast to therapeutic vaccines, where high affinity peptides are needed to overcome self-tolerance. By inducing a broad range of CTLs, the chance of generation of escape mutants decreases, rendering a vaccine more effective [59, 60]. Some successful phase I clinical trials describing influenza peptide vaccines capable of inducing T cell responses have been reported [9, 10]. However, these vaccines consist of long peptides and are mostly based on immunodominant epitopes, which might not be the best epitopes to induce a response to since there are indications that these epitopes overrule other T cell responses [57, 61].

We have shown for six influenza epitopes, all with different characteristics, that it is possible to improve their MHC binding affinity and that the immunogenicity of the three HLA-A*0201 epitopes could be improved considerably. Furthermore, by improving binding of HLA-A*0301-specific peptides we have shown that it is possible to target alleles other than HLA-A*0201, which is essential for broad population coverage. In order to enhance immunogenicity and efficacy of short peptides for T cell-targeted vaccines as used in our studies it is necessary to include adjuvants and to include a broader range of peptides. Our results illustrate the potential of inducing responses to otherwise subdominant epitopes by modification of amino acid residues and enhancing binding affinity. Especially since there are indications that inducing a broad response is more efficacious, our approach provides a promising method to induce responses to a larger range of epitopes [61].

## Supporting Information

S1 Fig Epitope MHC specificity control experiment in C57BL/6 mice. (DOCX)

S2 Fig Flow cytometry dot plots showing IFN-γ-positive CD4^+^ T cells of HLA-A2^+^ transgenic mice. (DOCX)

S1 Table HLA-A*0201 binding of CPLs of influenza epitopes; GILGFVFTL (M1_58-66_), FMYSDFHFI (PA_46-54_) and (C) NMLSTVLGV (PB1_413-421_). (XLSX)

S2 Table Heat map representation of Table 1. FP binding scores of selected HLA-A*0201 peptides.
(XLSX)

S3 Table GILG, FMY and NML specific CD8^+^ T cell responses after stimulation with CPLs in in vitro and ex vivo screening models. (XLSX)

S4 Table Heat map representation of Table 3. FP binding scores HLA-A*0301 peptides.
(XLSX)
