# Supplementary material for: Chemical Modification of Influenza CD8+ T-Cell Epitopes Enhances Their Immunogenicity Regardless of Immunodominance
Source: PLoS One. 2016 Jun 22;11(6):e0156462. doi: 10.1371/journal.pone.0156462 (PMC4917206; doi:10.1371/journal.pone.0156462)
Supplement: S1 Fig — (DOCX) [file pone.0156462.s001.docx]

**

**

**S1 Fig:** **Epitope MHC specificity control experiment in C57BL/6 mice.**

C57BL/6 and HLA-A2 tg mice were vaccinated with a dose of 75 nmol of WT peptides **(A)** or CPLs **(B)** on days 0 and 21. Two weeks later spleen cells were isolated and stimulated O/N with 0.1 nmol of WT peptides or corresponding CPLs. WT GILG and WT FMY (n=2), WT NML in HLA-A2 tg mice (n=5), WT NML in B6 (n=7), G1 and F5 (n=4), N53 HLA-A2 tg (n=6), C57BL/6 (n=7). No responses were detected in C57BL/6 mice vaccinated with WT peptides. However, C57BL/6 mice did respond to F5, although responses of HLA-A2 transgenic mice were higher. All WT and CPLs induced responses in the HLA-A2 tg mice. Bars are min to max, with line at mean.
