# Supplementary material for: Chemical Modification of Influenza CD8+ T-Cell Epitopes Enhances Their Immunogenicity Regardless of Immunodominance
Source: PLoS One. 2016 Jun 22;11(6):e0156462. doi: 10.1371/journal.pone.0156462 (PMC4917206; doi:10.1371/journal.pone.0156462)
Supplement: S2 Fig — (DOCX) [file pone.0156462.s002.docx]

**
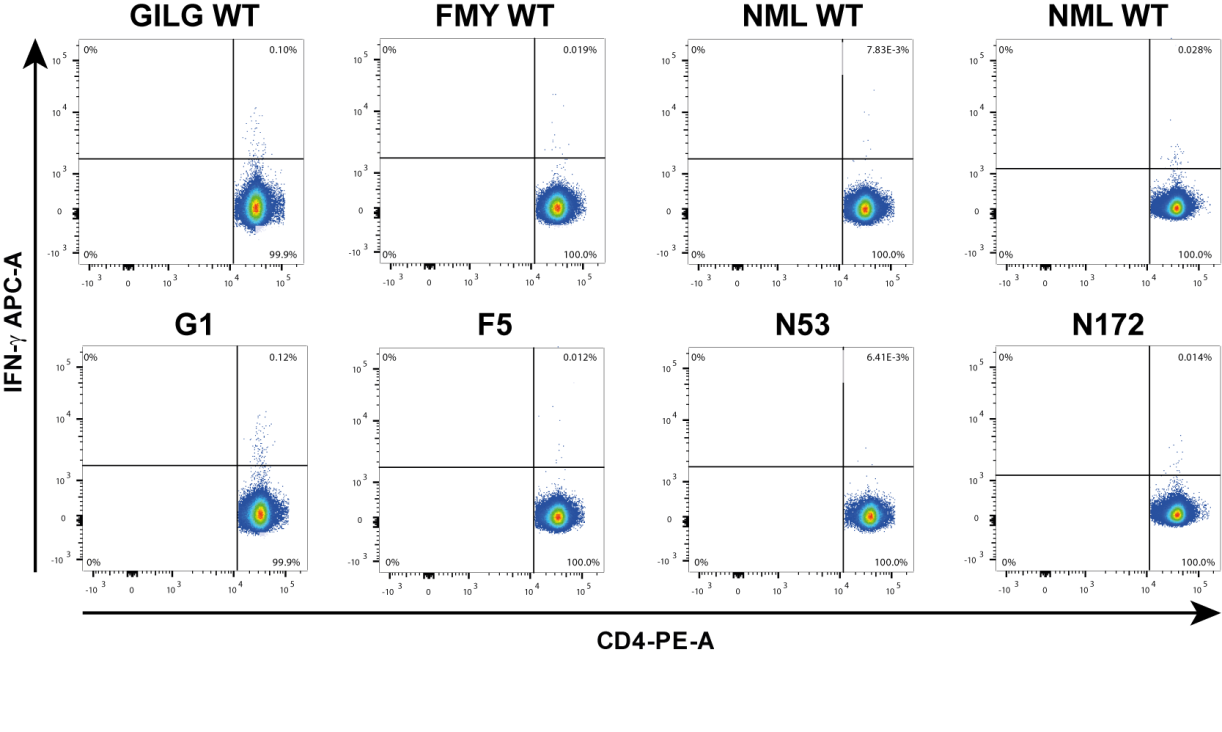
**

**S2 Fig: Flow cytometry dot plots showing IFN-γ-positive CD4^+^ T cells of HLA-A2^+^ transgenic mice.**

Dot plots show IFN-γ production by CD4^+^ T cells of mice vaccinated with 75 nmol of either WT peptide or CPL (G1, F5, N53 and N172). Spleen cells were stimulated for 16 hours with 0.1 nmol/well WT peptide. Highest responders of each group are shown. Although for GILGFVFTL some background staining is visible, these dot plots show that CD4^+^ T cells did not produce IFN-γ in response to peptide restimulation, indicating that the enhanced IFN-γ production measured in the ELISpot assay was indeed produced by CD8^+^ T cells and not CD4^+^ T cells.
